# Revisiting Frank–Starling: regulatory light chain phosphorylation alters the rate of force redevelopment (*k*_tr_) in a length‐dependent fashion

**DOI:** 10.1113/JP272441

**Published:** 2016-07-24

**Authors:** Christopher N. Toepfer, Timothy G. West, Michael A. Ferenczi

**Affiliations:** ^1^ Molecular Medicine SectionNational Heart and Lung Institute, Imperial College LondonLondonUK; ^2^ Laboratory of Molecular PhysiologyNHLBI, National Institutes of HealthBethesdaMDUSA; ^3^ Structure & Motion LaboratoryRoyal Veterinary College LondonNorth MymmsUK; ^4^ Lee Kong Chian School of MedicineNanyang Technological UniversitySingaporeSingapore

**Keywords:** cardiac muscle, force redevelopment, muscle contraction, phosphorylation, regulatory light chain

## Abstract

**Key points:**

- Regulatory light chain (RLC) phosphorylation has been shown to alter the ability of muscle to produce force and power during shortening and to alter the rate of force redevelopment (*k*_tr_) at submaximal [Ca^2+^].
- Increasing RLC phosphorylation ∼50% from the *in vivo* level in maximally [Ca^2+^]‐activated cardiac trabecula accelerates *k*_tr_.
- Decreasing RLC phosphorylation to ∼70% of the *in vivo* control level slows *k*_tr_ and reduces force generation.
- *k*_tr_ is dependent on sarcomere length in the physiological range 1.85–1.94 μm and RLC phosphorylation modulates this response.
- We demonstrate that Frank–Starling is evident at maximal [Ca^2+^] activation and therefore does not necessarily require length‐dependent change in [Ca^2+^]‐sensitivity of thin filament activation.
- The stretch response is modulated by changes in RLC phosphorylation, pinpointing RLC phosphorylation as a modulator of the Frank–Starling law in the heart.
- These data provide an explanation for slowed systolic function in the intact heart in response to RLC phosphorylation reduction.

**Abstract:**

Force and power in cardiac muscle have a known dependence on phosphorylation of the myosin‐associated regulatory light chain (RLC). We explore the effect of RLC phosphorylation on the ability of cardiac preparations to redevelop force (*k*_tr_) in maximally activating [Ca^2+^]. Activation was achieved by rapidly increasing the temperature (temperature‐jump of 0.5–20ºC) of permeabilized trabeculae over a physiological range of sarcomere lengths (1.85–1.94 μm). The trabeculae were subjected to shortening ramps over a range of velocities and the extent of RLC phosphorylation was varied. The latter was achieved using an RLC‐exchange technique, which avoids changes in the phosphorylation level of other proteins. The results show that increasing RLC phosphorylation by 50% accelerates *k*_tr_ by ∼50%, irrespective of the sarcomere length, whereas decreasing phosphorylation by 30% slows *k*_tr_ by ∼50%, relative to the *k*_tr_ obtained for *in vivo* phosphorylation. Clearly, phosphorylation affects the magnitude of *k*_tr_ following step shortening or ramp shortening. Using a two‐state model, we explore the effect of RLC phosphorylation on the kinetics of force development, which proposes that phosphorylation affects the kinetics of both attachment and detachment of cross‐bridges. In summary, RLC phosphorylation affects the rate and extent of force redevelopment. These findings were obtained in maximally activated muscle at saturating [Ca^2+^] and are not explained by changes in the Ca^2+^‐sensitivity of acto‐myosin interactions. The length‐dependence of the rate of force redevelopment, together with the modulation by the state of RLC phosphorylation, suggests that these effects play a role in the Frank–Starling law of the heart.

## Key points

- Regulatory light chain (RLC) phosphorylation has been shown to alter the ability of muscle to produce force and power during shortening and to alter the rate of force redevelopment (*k*_tr_) at submaximal [Ca^2+^].
- Increasing RLC phosphorylation ∼50% from the *in vivo* level in maximally [Ca^2+^]‐activated cardiac trabecula accelerates *k*_tr_.
- Decreasing RLC phosphorylation to ∼70% of the *in vivo* control level slows *k*_tr_ and reduces force generation.
- *k*_tr_ is dependent on sarcomere length in the physiological range 1.85–1.94 μm and RLC phosphorylation modulates this response.
- We demonstrate that Frank–Starling is evident at maximal [Ca^2+^] activation and therefore does not necessarily require length‐dependent change in [Ca^2+^]‐sensitivity of thin filament activation.
- The stretch response is modulated by changes in RLC phosphorylation, pinpointing RLC phosphorylation as a modulator of the Frank–Starling law in the heart.
- These data provide an explanation for slowed systolic function in the intact heart in response to RLC phosphorylation reduction.

**Abbreviations**
CF: conversion factor
DR: duty ratio
*f*: rate constant of cross‐bridge attachment (s^−1^)
*F*: force produce by an individual cross‐bridge (pN)
*F*_1.85_: force of a maximally activated trabecula at a sarcomere length of 1.85 μm (kN m^–2^)
*F*_1.90_: force of a maximally activated trabecula at a sarcomere length of 1.90 μm (kN m^–2^)
*F*_1.94_: force of a maximally activated trabecula at a sarcomere length of 1.94 μm (kN m^–2^)
*F*_iso_: isometric force (kN m^–2^)
FL s^–1^: trabecula shortening velocity (trabecula lengths s^–1^)
*F*_ramp_: force sustained during a shortening ramp at a steady velocity (kN m^–2^)
*F*_rec_: force recovered in isometric conditions after the end of protocol (kN m^–2^)
*g*: rate constant of cross‐bridge detachment (s^−1^)
*k*_1.85_: rate of force redevelopment (*k*_tr_), at a sarcomere length of 1.85 μm (s^−1^)
*k*_1.90_: rate of force redevelopment (*k*_tr_), at a sarcomere length of 1.90 μm (s^−1^)
*k*_1.94_: rate of force redevelopment (*k*_tr_), at a sarcomere length of 1.94 μm (s^−1^)
*k*_tr_: rate of force redevelopment (s^–1^)
*N*: number available cross‐bridges for active cycling
RLC: regulatory light chain
SL: sarcomere length (μm)

## Introduction

Contraction in striated muscle is driven by interactions between actin and myosin, and is controlled by the availability of free Ca^2+^ to bind the thin filament regulatory complex (troponin complex). This ‘on/off’ mechanism in striated muscle determines the availability of myosin binding sites on actin (thin filament activation) (Moss *et al*. 2004). In the presence of calcium, these sites are available and cyclical interactions between actin and myosin occur, producing force and shortening. In addition, RLC phosphorylation level plays a role in modulating the ability of cross‐bridges to produce force, especially under load (Toepfer *et al*. 2013). What has not yet been well established is the ability of RLC phosphorylation to alter the kinetics of force redevelopment (*k*_tr_) (not created by stretch activation) (Stelzer *et al*. 2006 *a*; Stelzer *et al*. 2006 *b*) in cardiac tissue. Force redevelopment was described in 1957 by Huxley using a simple two‐state model, which described *k*_tr_ as the sum of the forward (*f*), and backward (*g*) rate constants for the transition into (and out of) the load bearing acto‐myosin states, respectively (Huxley, 1957). Thus, processes that affect *f* or *g* modify the amplitude of force and the rate of force redevelopment. The amount of isometric force produced (*F*_iso_) is given by:

F iso =F·N·f/f+g

where *F* is the force per cross‐bridge, *N* is the total number of cross‐bridges in the filament overlap region and *f*/(*f* + *g*) is the proportion of cross‐bridges in force generating states.

Subsequent studies have shown that the rate constants *f* and *g* are complex terms, dependent on transitions between multiple biochemical intermediates (Pate & Cooke, 1986; Regnier *et al*. 1998). Although the two‐state model does not describe the transient force response following step changes in length, it is a useful tool for making comparisons between treatment groups (Huxley & Simmons, 1971; Podolsky *et al*. 1976); for example, the rate of ADP release from the myosin active site was identified as both strain sensitive and a major determinant of *g* in striated muscle (Nyitrai & Geeves, 2004; Greenberg *et al*. 2014). The rate of *f* is dependent on thin filament activation (co‐operative activation) (Bremel & Weber, 1972; Brenner, 1988; Araujo & Walker, 1994; Wolff *et al*. 1995), which in effect alters the availability of actin binding sites to which myosin heads may bind, a factor also considered to be influenced by lattice expansion or shrinkage and by regulatory light chain (RLC) phosphorylation, shifting the myosin heads closer to the thin filament by addition of a negative charge to the neck region (Barany *et al*. 1980; Ueno & Harrington, 1981; Levine *et al*. 1996; Colson *et al*. 2010). Previous studies investigating the effect of RLC phosphorylation on *k*_tr_ and on the stretch activation response in rat cardiac trabeculae were performed using relatively long sarcomere lengths (SL) (2.2–2.3 μm), in the presence of either limiting or maximal [Ca^2+^], with conflicting results (Morano *et al*. 1995; Olsson *et al*. 2004; Stelzer *et al*. 2006 *b*; Colson *et al*. 2010). Work on skeletal muscle in submaximal [Ca^2+^] clearly identified RLC phosphorylation as a determinant of *k*_tr_ (Metzger *et al*. 1989; Sweeney & Stull, 1990). However, the role that RLC phosphorylation plays in regulating *k*_tr_ in cardiac muscle after shortening and at physiological sarcomere lengths observed during systole (1.91–1.68 μm) (Sonnenblick *et al*. 1967) is unknown.

By carrying out experiments in the presence of saturating [Ca^2+^], the effect of calcium‐dependent activation is eliminated such that any changes in *k*_tr_ depend on the RLC phosphorylation levels and not on the calcium regulatory complex (de Tombe & Stienen, 2007). The role of RLC phosphorylation warrants investigation because the observed changes in RLC phosphorylation levels in myocardium, above or below basal levels, change both power output and force production of the tissue (Toepfer *et al*. 2013) Additionally, there is a close association between RLC phosphorylation and myocardial function, such as in disease models, both acquired and inherited (Sheikh *et al*. 2014).

In the present study, we use an *in vitro* RLC exchange protocol to alter RLC phosphorylation level in rat cardiac ventricular trabeculae, without affecting phosphorylation of other sarcomeric constituents (Toepfer *et al*. 2013). Activation of the permeabilized trabeculae is achieved by temperature‐jump to ensure rapid and homogeneous activation and thereby avoiding the confounding effects of Ca‐diffusion into the core of the trabeculae. We measure *k*_tr_ using a step release protocol at three physiological RLC phosphorylation levels that are observed in health and disease (Sheikh *et al*. 2014). This provides a measurement of absolute force redevelopment when all cross‐bridges are primed to begin actively cycling after being forcibly detached by a rapid release. Measurements are carried out at three sarcomere lengths (SL, 1.94, 1.90 and1.85 μm), with the shortest being that encountered during late systole *in vivo* (Guccione *et al*. 1997; Hanft *et al*. 2008). We use release‐ramp protocols to elicit force redevelopment to measure *k*_tr_ over a range of physiological shortening velocities that the intact myocardium would encounter during systole. This technique allows us to assess *k*_tr_ with unsynchronized cross‐bridges populating a variety of cross‐bridge states, similar to that occurring in the shortening myocardium during systole. Hence, we aim to advance our mechanistic understanding of how RLC phosphorylation alters *k*_tr_, independently of calcium‐dependent co‐operative activation in cardiac muscle.

## Methods

### Ethical approval

All animal procedures were carried out in accordance with the Guide for the Care and Use of Laboratory Animals, published by the United States National Institutes of Health, under assurance number A5634‐01. Cervical dislocation was performed in accordance with Schedule 1 of the UK Home Office Animals (Scientific Procedures) Act 1986.

### Trabeculae preparation

Female Sprague–Dawley rats (fed *ad libitum*) weighing 250–350 g were anaesthetized using isofluorane inhalation, until unconscious. Rapid cervical dislocation was followed by removal of the heart by abdominal and subsequent thoracic resection. Hearts were excised with intact aortic arches for cannulation and retrograde perfusion with oxygenated ice‐cold Krebs–Henseleit solution (composition in mm: 119 NaCl, 4.7 KCl, 0.94 MgSO_4_, 1 CaCl_2_, 1.2 KH_2_PO_4_, 25 NaHCO, 11.5 glucose and 30 2,3‐butanedione monoxime, in addition to 12 units ml^–1^ of heparin). Left ventricular trabeculae (1–2 mm in length and 100–300 μm in width) were excised from the resected left ventricle. Trabeculae ends were then crimped in T‐clips and pinned at resting length on Sylgard for chemical permeabilization using relaxing solution (composition in mm: 60 TES, 8.66 MgCl_2_, 20 EGTA, 5.43 Na_2_ATP, 10 glutathione and 33.71 potassium propionate at pH 7.4) for 30 min at 5°C, incorporating 2% Triton X‐100. Trabeculae were stored post‐permeabilization at –20°C in 50% relaxing solution with 50% glycerol and protease inhibitors (4 mg l^–1^ leupeptin, 10 mm phenylmethanesulphonyl fluoride and 50 mg l^–1^ trypsin inhibitor). Trabeculae were stored for no longer that 3 days prior to experimentation.

### RLC exchange

The methods for exchange and for measuring the extent of exchange have been described previously (Toepfer *et al*. 2013). Briefly, recombinant cardiac RLC from *Rattus norvegicus* was expressed, purified and dephosphorylated or phosphorylated *in vitro* using shrimp alkaline phosphatase or smooth muscle myosin light chain kinase, respectively (Toepfer *et al*. 2013). RLCs with known phosphorylation levels (0.1 mol Pi mol^–1^ RLC, 0.5 mol Pi mol^–1^ RLC and 1.1 mol Pi mol^–1^ RLC) were exchanged for native RLC in permeabilized trabeculae using an exchange buffer (composition in mm: 5 ATP, 5 EGTA, 5 EDTA, 10 imidazole, 150 potassium propionate, 10 KH_2_PO_4_, 5 DTT and 0.5 trifluoperazine at pH 6.5). Exchange was performed with 0.6 mg ml^–1^ RLC on the experimental set‐up at a sarcomere length of 2.1 μm at 0.5°C for 45 min. The extent of RLC phosphorylation in the trabeculae was calculated from the measured time course of RLC exchange (Toepfer *et al*. 2013). Exchange protocols were adjusted to obtain three RLC phosphorylation levels in trabeculae. These were increased phosphorylation (phosphorylated) with a normalized RLC phosphorylation of 1.5 ± 0.1 (∼0.66 mol Pi mol^–1^ RLC) compared to native where the extent of phosphorylation is 0.44 mol Pi mol^–1^ RLC, reduced phosphorylation of 0.7 ± 0.05 (∼0.31 mol Pi mol^–1^ RLC) and control exchange mimicking native RLC phosphorylation levels of 1.1 ± 0.1 (Toepfer *et al*. 2013).

### Trabeculae mechanics

After exchange, the ends of the trabeculae were attached to the apparatus with shellac and the sarcomere length was adjusted to 2.1 μm, using the first‐order sarcomere diffraction line visualized by illumination with a HeNe laser. Contraction was triggered by a temperature‐jump method where the preparation cycles through a series of solutions at high (20°C) and low (0.5°C) temperatures to activate and relax muscle contraction, respectively (Toepfer *et al*. 2013). The protocol consists of: (1) relaxing solution at 0.5°C, where no force is seen, apart from that as a result of resting tension; (2) pre‐activation solution 0.5°C; (3) activating solution 0.5°C, where, at this temperature, only a little force develops (<5% of that at 20°C); (4) activating solution 20°C (shortening protocols are applied once isometric plateau is reached); and (5) and relaxing solution 20°C, where force decreases back to zero. The preparation is then returned to relaxing solution at 0.5°C and the cycle is repeated. Solution compositions are shown in Table 1. The sarcomere length was rechecked at the end of each experiment to ensure that no slippage had occurred. Each trabecula went through up to nine activation cycles without showing significant deterioration of force. Each trabecula was tested at several shortening ramp velocities (see below).

**Table 1:** Experimental solutions

	Relaxing	Pre‐activating	Activating
	(pH 7.1)	(pH 7.1)	(pH 7.1)
Tes	100	100	100
MgCl_2_	7.8	6.8	6.5
Na_2_ATP	5.7	5.7	5.7
EGTA	25	0.1	0
GLH	20	20	20
HDTA	0	24.9	0
CaEGTA	0	0	25

All concentrations are in mm. pH was set for the specific temperature of the solution used. GLH, glutathione; HDTA, 1,6‐diaminohexane‐*N*,*N*,*N′*,*N′*‐tetraacetic acid.

Two mechanical perturbation protocols were used in the experiments described in the present study. The first protocol consists of a rapid (step release) reduction in trabecula length, applied during the plateau of isometric force development at 20°C, starting at a sarcomere length of 2.1 μm. The speed and amplitude of the applied release is such that the trabecula becomes momentarily slack. Following the release, force re‐develops. Force re‐development is characterized in terms of the amplitude of force regained and the rate constant describing the speed of force redevelopment.

The second protocol, ‘release‐ramp’, consists of a small and fast step release, followed by a slower decrease in trabecula length at a steady shortening velocity at 20°C. The step release unloads the instantaneous elasticity of the preparations and the ramp shortening allows the trabecula to reach a steady tension, which is characteristic for the ramp velocity, thus defining the force–velocity relationship. The effects of RLC phosphorylation on the force–velocity relationship have been reported previously (Toepfer *et al*. 2013). In the present study, we use the protocol to explore the redevelopment of force following the period of steady shortening. As in the ‘step release’ protocol, two parameters are measured: the amount of force regained and the rate of force redevelopment. Details of the two protocols are given below.

### Step release protocol

**Figure 1:**
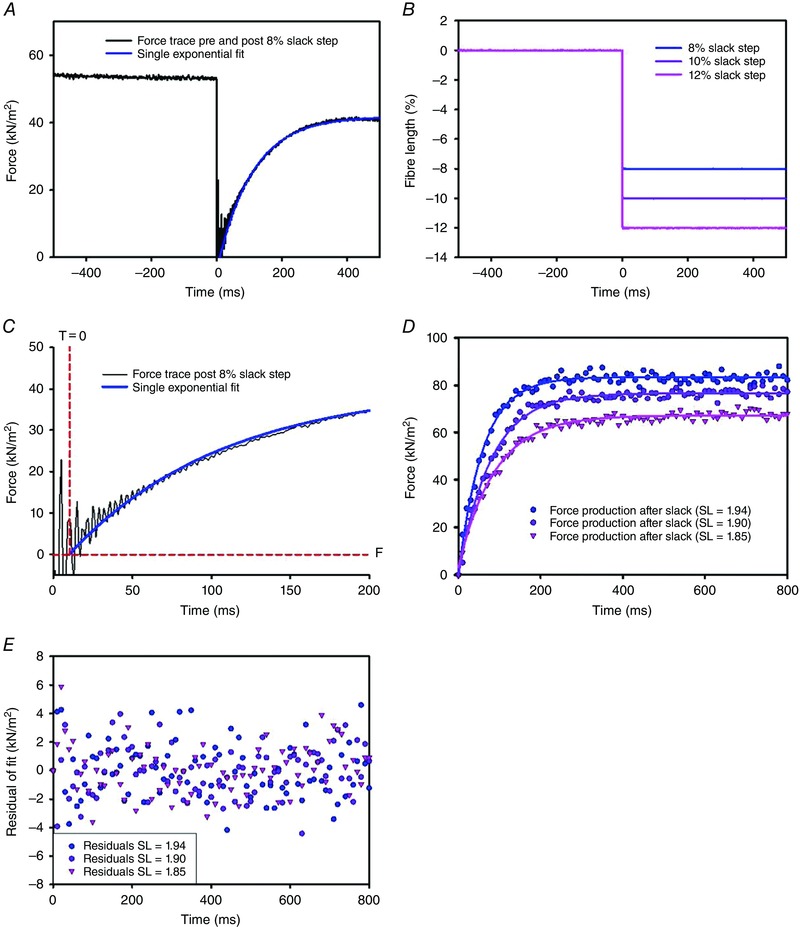
**Step‐release protocol on control trabecula** *A*, time course of force changes (black) of a trabecula following temperature‐jump activation. Isometric force (*F*_iso_) is reached before an 8% step release is applied. Force recovery after the release is fit with a single exponential (Blue & McMurray, 2005). *B*, representative motor output traces showing the step protocols for step release protocols of three different amplitudes. *C*, expanded force trace directly after step‐release. Time zero is set to be the time after the end of the step release when force deviated from zero (*F* = 0). *D*, representative force recoveries observed after step releases of variable amplitude leading to different sarcomere lengths after the end of the step release. Each plot is fit with a single exponential (continuous lines). *E*, plot of residuals for each slack protocol performed in (*D*).

Each trabecula preparation developed peak isometric force at a sarcomere length of 2.1 μm. Rapid length changes, which were faster than the maximal shortening velocity of the unloaded preparation, were applied to the trabeculae, with shortening amplitudes of –8% (end‐point sarcomere length: 1.94 μm), –10% (1.90 μm) and –12% (1.85 μm) of the initial sarcomere length (2.1 μm) during activation. Isometric force redeveloped at each nominal sarcomere length, with an amplitude denoted as *F*_1.94_, *F*_1.90_, and *F*_1.85_. A representative force trace for a –8% step is shown in Fig. 1 *A*. Figure 1 *B* shows the length transducer signal for shortenings at each of the three amplitudes. Each force recovery time‐course was fit with a two‐parameter exponential:

F=Fr ec (1−e−k tr (t))

where *F*_r_ec is the steady‐state force recovered post protocol, *F* is the force at time *t* and *k*_tr_ is the rate of force redevelopment post protocol.

The exponential was applied from *t* = 0, which was the time at which force was discernibly above zero (*F* = 0). This time point was when the slack had been taken up by active shortening of the trabeculae to the new lower sarcomere length (Fig. 1 *C*). Figure 1 *D* shows the exponential fit to three representative traces for force re‐development by the same trabecula at three sarcomere lengths. The fitted rate constants were *k*_1.94_ = 10 s^–1^, *k*_1.90_ = 9 s^–1^ and *k*_1.85_ =_ _8 s^–1^. Figure 1 *E* shows the scatter of residuals for the fit to plots in Fig. 1 *D*. This protocol was applied to each trabecula at each RLC phosphorylation level.

### Release‐ramp protocol

**Figure 2:**
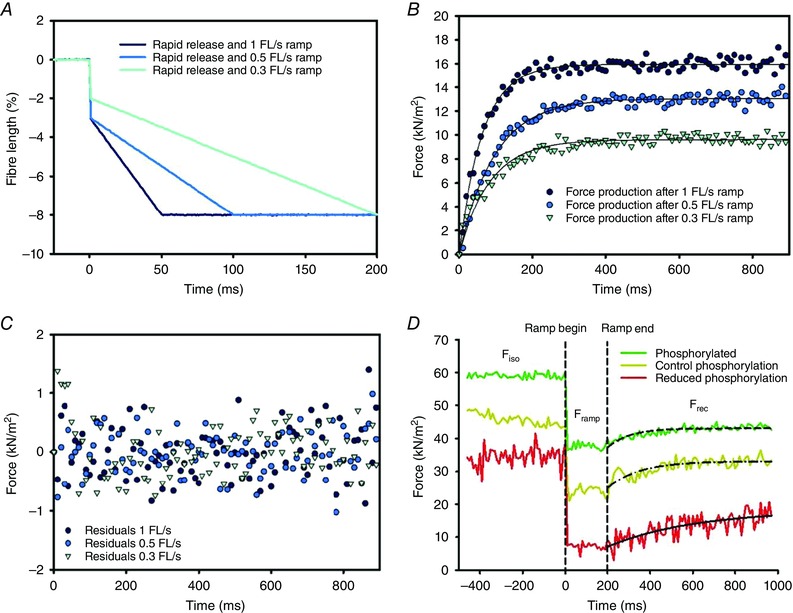
**Release‐ramp protocols** *A*, time course of length changes of varying speed and amplitude applied to trabeculae by the length transducer. The differences between 0.3, 0.5 and 1 FL s^–1^ release velocities are seen. *B*, time course of force recovery (*F*_rec_) after release‐ramp protocols of different velocities. Each force recovery is fit with a single exponential. *C*, plot of residuals for each release ramp protocol shown in (*B*). *D*, time course of force changes from three trabeculae at differing RLC phosphorylation levels, induced by the slowest ramp shown in (*A*). The force signals show that the force level during the fixed velocity ramp (*F*_ramp_) depends on the RLC phosphorylation level. Phosphorylation also affects the ability of trabeculae to recover force after the end of the ramp‐release (*F*_rec_).

The release‐ramp protocols were designed to determine the force‐velocity relationship in trabeculae for a range of RLC phosphorylation levels. A byproduct of these protocols is the time‐course of force recovery following the period of shortening, which we find to be sensitive to RLC phosphorylation levels, providing information about the kinetics of actomyosin interactions. Isometric force was allowed to develop at a sarcomere length of 2.1 μm. At the plateau of isometric force, the length transducer applied a rapid shortening to the trabecula, followed by a slower shortening (Fig. 2 *A*). Depending on the ramp velocity, the amplitude of the initial rapid release length was altered to minimize series elasticity during ramp, which resulted in a relatively stable force signal during ramp release (Fig. 2 *D*). This protocol provides a reliable method for determining the experimental relationship between force and velocity (Curtin *et al*. 1998). Ramp velocities of 1, 0.5 and 0.3 FL s^–1^ were applied, where the velocity is expressed as fibre lengths per second (FL s^–1^), with fibre length being the length of the trabecula between the T‐clips when held at a sarcomere length of 2.1 μm. The parameters describing the length change protocols are given in Table 2.

**Table 2:** **Experimental release‐ramp protocol for each ramp velocity**

Ramp release	Release amplitude	Ramp amplitude
(FL s^–1^)	(% trabecula length)	(% trabecula length)
1.0	3%	5%
0.5	3%	5%
0.3	2%	6%

All protocols released to a maximum of 8% of initial muscle length. The breakdown of the release‐ramp amplitudes for each velocity protocol is shown

Each protocol reduced sarcomere length by ∼8% to 1.94 μm. Figure 2 *B* shows representative traces of the force developing after the end of the ramp, starting at a sarcomere length of 1.94 μm. Isometric force reached after each ramp is given as *F*_1_, *F*_0.5_ and *F*_0.3_, where the subscript denotes the shortening velocity during the release ramp. Figure 2 *C* shows the scatter of residuals for the exponential fit to the force data in Fig. 2 *B*. Figure 2 *D* shows the force records during the release‐ramp protocols including the subsequent force redevelopment, for trabeculae incorporating RLC at three different phosphorylation levels. The higher the phosphorylation level of the sample, the higher the force attained during initial force development at a sarcomere length of 2.1 μm (*F*_iso_) and during ramp (*F*_ramp_). The fit to the force recovery records after ramp shortening gave estimates for the rate constant describing the time‐course of force redevelopment. The rate constants were obtained for force recovery following ramps at three different velocities (see above) and are therefore denoted *k*_1_, *k*_0.5_ and *k*_0.3_ for the three ramp velocities of 1, 0.5 and 0.3 FL s^–1^. These rate constants were calculated for each of the three phosphorylation states.

### Modelling a two‐state cross‐bridge cycle

The model considers:

R<−>A

where *R* is the relaxed state and *A* is the active, force‐generating state. The rate constants *f* and *g* determine transition into the force‐generating state and into the relaxed state, respectively.

**Figure 3:**
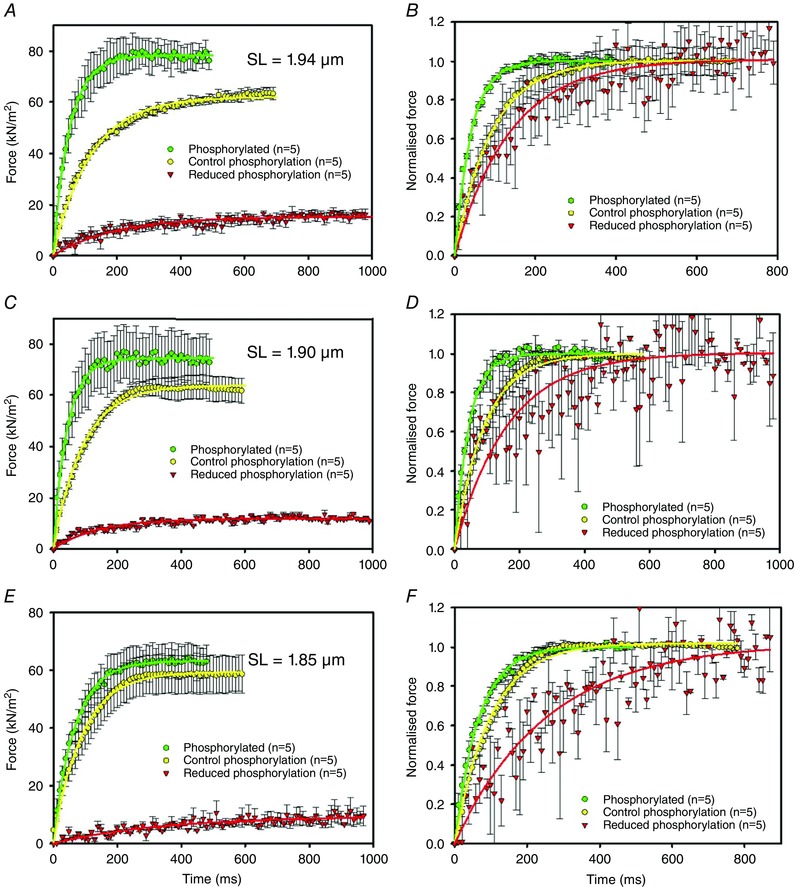
**The effect of step release protocol on force redevelopment at three RLC phosphorylation levels** *A*, averaged time course of force recovery *F*_rec_ at SL = 1.94 μm. *B*, same records as in (*A*) after normalization for isometric force reached after force recovery. *C*, averaged time course of force recovery *F*_rec_ at SL = 1.90 μm. *D*, same records as in (*C*) after normalization. *E*, averaged time course of force recovery *F*_rec_ at SL = 1.85 μm. *F*, same records as in (*E*) after normalization. Each trace in (*B*), (*D*) and (*E*) is fitted with an exponential function to determine *k*_tr_. All data points are the mean ± SEM (*n = *5) and fit by a single exponential.

In such a model, the amount of force at any time (*t*) is given by a single exponential equation as:

$$Y=\left[\mathrm{fxN}\;/(f+g)\right]\times \left[1-e^{\left(-(f+g)\times T\right)}\right]$$

where *Y* is the amount of force generating cross‐bridges, *N* is the total concentration of cross‐bridges and *T* is time. The fraction of actin‐attached cross‐bridges is given by *Y*/*N*, which is also referred to as the duty ratio (DR). According to this model, the rate constant describing force generation is (*f* + *g*). The rate of force development observed in the experiments described in the present study thus give estimates for (*f* + *g*) under various conditions of RLC phosphorylation and sarcomere length. The observed time course of force generation is well described by a single exponential process (Fig. 1 *D* and *E* and Figs 2 and 3).

For any given muscle type at full isometric activation, the DR is constant, which was approximated as 50% at control phosphorylation (He *et al*. 2000). This is 100 μm of myosin heads out of the ∼200 μm available to form cross‐bridges. To convert DR into a force measurement, we consider an empirical conversion factor (CF) given by:

CF =F iso / DR

The CF was assumed to be constant between RLC phosphorylation levels.

This allowed the calculation of DR for each sarcomere length and RLC phosphorylation level. Knowing *k*_tr_, *F*_iso_ and CF allowed for the calculation of *g* and *f* as:

g= CF −F iso ×k tr / CF

f=k tr −g

Calculating DR for each RLC phosphorylation level and SL allowed the approximation of force per cross‐bridge (*F*_XB_), which is given by:

F XB =F iso / total  heads × DR

*F*_XB_ (in pN) and the total number of heads were derived by assuming a constant concentration of 200 μm of myosin heads (Ferenczi *et al*. 1984).

The modelling results are shown in Table 3.

**Table 3:** Parameters from exponential fit and two state modelling of release protocol

	Phosphorylated (*n = *5)			Control phosphorylation (*n = *5)			Reduced phosphorylation (*n = *5)		
Sarcomere length (μm)	1.94	1.90	1.85	1.94	1.90	1.85	1.94	1.90	1.85
*F*_iso_	78 ± 7^†^	74 ± 8^†^	63 ± 6^†^	63 ± 5	60 ± 6	58 ± 7	16 ± 4	12 ± 3	8 ± 3
*k*_tr_ (s^–1^)	24.0 ± 1.8*, ^†^	22.2 ± 1.2^†^	14.9 ± 1.4^†^	11.0 ± 1.7*	10.7 ± 1.0	10.0 ± 0.4	7.2 ± 0.9*	6.1 ± 0.9	3.9 ± 1.0
Duty ratio	0.67 ± 0.05*, ^†^	0.59 ± 0.09^†^	0.50 ± 0.06^†^	0.50 ± 0.03*	0.48 ± 0.05	0.46 ± 0.07	0.13 ± 0.01*	0.09 ± 0.01	0.06 ± 0.02
*f* (s^–1^)	18.7 ± 1.4*, ^†^	12.9 ± 1.9^†^	7.5 ± 0.9^†^	5.5 ± 0.3	5.1 ± 0.5	4.6 ± 0.7	0.9 ± 0.1	0.6 ± 0.02	0.3 ± 0.1
*g* (s^–1^)	9.1 ± 0.7*, ^†^	9.1 ± 1.4^†^	7.5 ± 0.9^†^	5.5 ± 0.3	5.7 ± 0.5	5.4 ± 0.8	6.3 ± 0.6*	5.4 ± 0.2	3.7 ± 0.9
F_XB_ (pN)	3.2 ± 0.08	2.8 ± 0.16	2.8 ± 0.12	3.0 ± 0.07	3.1 ± 0.11	3.4 ± 0.19	3.1 ± 0.12	2.9 ± 0.07	3.5 ± 0.34

^*^Denotes a significant effect between SL 1.94 and 1.85 μm (*P* < 0.05).

^†^Denotesa significant effect of RLC phosphorylation within a SL length (*P* < 0.05).

### Statistical analysis

We hypothesized that: (i) RLC phosphorylation and (ii) sarcomere length affected *k*_tr_ at maximal calcium activation. Multiple comparisons between RLC phosphorylation groups and protocols were made using two‐way ANOVA to test experimental hypotheses. The hypotheses tested were (i) that SL affected *k*_tr_ and (ii) that RLC phosphorylation affected *k*_tr_. Appropriate *post hoc* pairwise multiple comparisons were made using Bonferroni adjusted *t* tests, with a statistical significance cut‐off of *P < *0.05. SigmaPlot (Systat Software Inc., Chicago, IL, USA) was used to perform statistical analysis. *N* is the number of trabeculae sampled for each protocol. Each treatment group comprised of five cardiac preparations.

## Results

### The effect of RLC phosphorylation on the rate of force redevelopment and force level after step release

**Figure 4:**
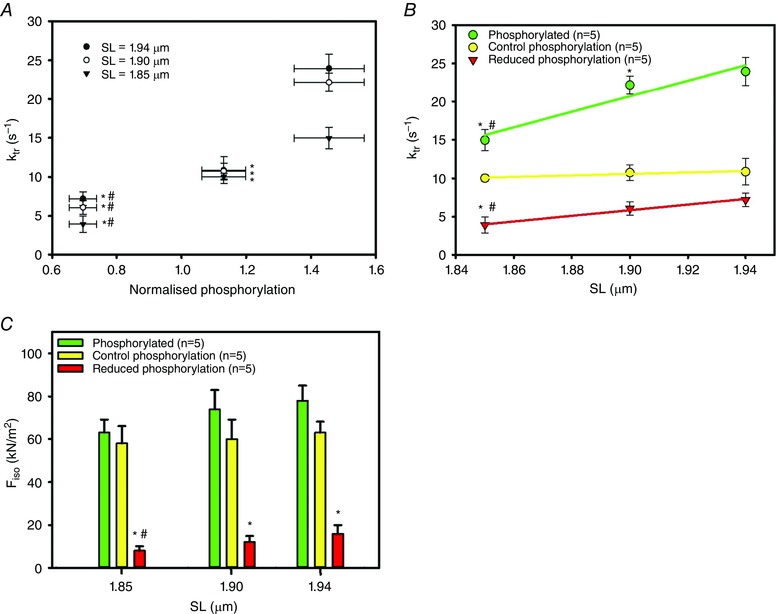
**The effect of RLC phosphorylation on k_tr_ following the step‐release protocol** *A*, plot of *k*_tr_ as a function of normalized RLC phosphorylation at three sarcomere lengths. Reduced phosphorylation is 0.69 ± 0.04, control phosphorylation is 1.13 ± 0.07 and phosphorylated is 1.45 ± 0.1. ^*^Significant difference compared to phosphorylated, where *P < *0.05. ^#^Significant difference compared to control phosphorylation where *P < *0.05. *B*, plot of *k*_tr_ as a function of sarcomere length (SL) for three different RLC phosphorylation levels. Data are the mean ± SEM. Linear regression is used to fit *k*_tr_ as a function of sarcomere length data (*n* = 5 for each point). The slopes of the regressions are 79 ± 14, 8 ± 0.2 and 26 ± 3 s^−1 ^μm^−1^ for phosphorylated, control phosphorylation and reduced phosphorylation, respectively (*n* = 5). Significance was achieved between each slope, where *P < *0.05. ^*^Significant difference compared to 1.94 μm, where *P < *0.05. ^#^Significant difference compared to 1.90 μm, where *P < *0.05. *C*, bar graph depicting *F*_iso_ for each RLC phosphorylation level at each sarcomere length. ^*^Significant difference compared to control phosphorylation (and phosphorylated) within SLs, where *P < *0.05. ^#^Significant difference compared to corresponding phosphorylation levels at 1.94 μm, where *P* ≤ 0.05.

Measuring force redevelopment from slack allows the assessment of force production from a state where many of the cross‐bridges will be synchronized in the unbound and weakly bound states (diastole). Therefore, inferences can be made about the attachment and detachment kinetics of myosin and how these parameters can be altered by SL, as well as RLC phosphorylation. Force redevelopment from slack at sarcomere lengths of 1.94 , 1.90 and 1.85 μm was measured for three different RLC phosphorylation levels (Fig. 3). These SL were used to minimize passive tension and to simulate the SL lengths that are transitioned through in late systole (Sonnenblick *et al*. 1967). For SL = 1.94 μm (Fig. 3 *A*), the extent of force recovery was higher at higher levels of RLC phosphorylation, and reduced by decreasing RLC phosphorylation. Trabeculae with control levels of RLC phosphorylation produced an isometric force at a SL of 1.94 μm (*F*_1.94_) of 63 ± 5 kN m^–2^ (Fig. 4 *C*). This was not significantly different from trabeculae with increased RLC phosphorylation (78 ± 7 kN m^–2^) but was higher than in trabeculae with reduced phosphorylation (16 ± 4 kN m^–2^; *P < *0.05). This was unsurprising because we have shown this phenomenon previously (Toepfer *et al*. 2013). The rate constant describing force redevelopment, with a starting sarcomere length of 1.94 μm (*k*_1.94_), was also affected by RLC phosphorylation. Unlike force, *k*_tr_ is affected in a dose‐dependent fashion by RLC phosphorylation where control levels of RLC phosphorylation show an intermediate rate of *k*_1.94_ 11 ± 1.7 s^–1^, to increased and reduced phosphorylation *k*_1.94_ 24 ± 1.8 and 7.2 ± 0.9 s^–1^ respectively, with both being significantly different from control (*P < *0.05) (Fig. 3 *B*). At the shorter sarcomere lengths of 1.90 and 1.85 μm, differences in isometric force between control trabeculae and phosphorylated trabeculae were not observed (Fig. 3 *C* and *E* and Fig. 4 *C*). However, in both instances, trabeculae with reduced RLC phosphorylation exhibited a significantly lower force when compared to control and phosphorylated samples at a SL of 1.94 μM (*P < *0.05). Phosphorylation level similarly affected the rate of force redevelopment at shorter sarcomere lengths in a manner the same as that for the longer starting sarcomere length of 1.94 μm (*P < *0.05) (Fig. 4). Therefore, the affect of RLC phosphorylation on *k*_tr_ is an independent modulator of *k*_tr_, which is not altered by SL in this range. Strikingly, at a sarcomere length of 1.85 μm, trabeculae with reduced RLC phosphorylation (70% of control) generated only a small amount of force (10–15%) compared to the values at control and 150% phosphorylation. (*P < *0.05). The reason for this effect is unclear. However, reducing RLC phosphorylation reduces the ability of muscle to generate force at full thin filament activation, indicating that either force per cross‐bridge or cross‐bridge formation is significantly hindered.

Normalizing to the isometric force at each sarcomere length highlights the effect of RLC phosphorylation on *k*_tr_ by allowing a direct comparison of the time‐course of force redevelopment at each phosphorylation level (Fig. 3 *B*, *D* and *F*). This highlights the role of RLC phosphorylation on force redevelopment at the three physiological sarcomere lengths tested (Fig. 4 *A*).

Additionally, for each RLC phosphorylation level, *k*_tr_ is slower at shorter sarcomere lengths (Fig. 4 *B*). The gradients of linear regressions applied to the data in Fig. 4 *B* were 79 ± 14, 8 ± 0.2 and 26 ± 3 s^−1 ^μm^−1^ for phosphorylated, control phosphorylation and reduced phosphorylation, respectively (*n* = 5). Statistical significance was observed between each slope (*P < *0.05).

### The effect of RLC phosphorylation on the rate of force production and extent of redeveloped force after release‐ramp protocols

**Figure 5:**
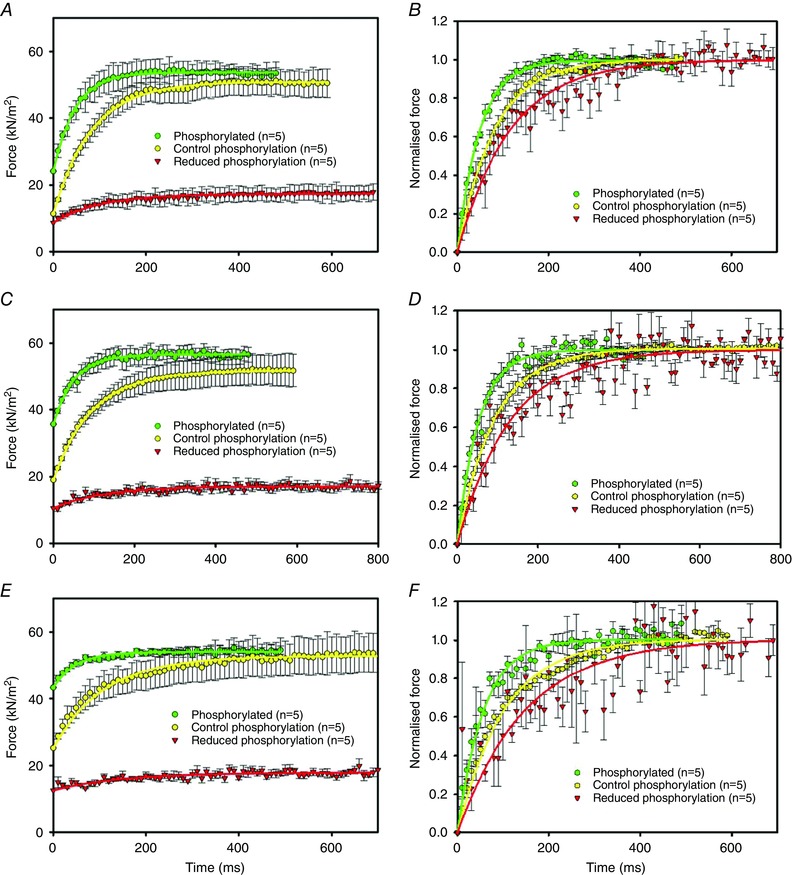
**The effect of RLC phosphorylation levels on *k*_tr_ in release‐ramp protocols on force redevelopment at three RLC phosphorylation levels** *A*, time course of force redevelopment *F*_rec_ after shortening at 1 FL s^–1^. *B*, the same records as in (*A*) after normalization. *C*, time course of force redevelopment *F*_rec_ after shortening at 0.5 FL s^–1^. *D*, The same records as in (*C*) after normalization. *E*, time course of force redevelopment *F*_rec_ after shortening at 0.3 FL s^–1^. *F*, the same records as in (*E*) after normalization. Each trace in (*B*), (*D*) and (*E*) is fitted with an exponential function to determine *k*_tr_. All data points are the mean ± SEM (*n = *5) and fit by single exponential.

**Figure 6:**
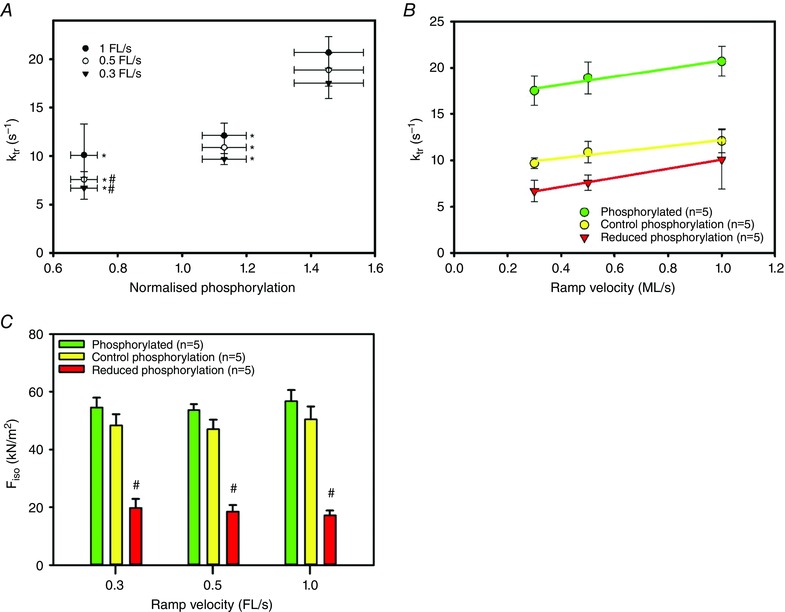
**The effect of RLC phosphorylation on k_tr_ following the release‐ramp protocol** *A*, the rate of force redevelopment *k*_tr_ as a function of normalized RLC phosphorylation at SL = 1.94 μm following ramp shortening at three velocities. Reduced phosphorylation is 0.69 ± 0.04, control phosphorylation is 1.13 ± 0.07 and phosphorylated is 1.45 ± 0.1. ^*^Significant difference compared to phosphorylated, where *P < *0.05. ^#^Significant difference compared to control phosphorylation, where *P < *0.05 *B*, the rate of force redevelopment *k*_tr_ as a function of shortening velocity for three different RLC phosphorylation levels. Data are shown as the mean ± SEM (*n = *5). There are no significant differences within RLC phosphorylation levels. Linear regression is used to fit *k*_tr_ as a function of ramp velocity data. There are no significant differences between the slopes. *C*, bar graph depicting *F*_iso_ for each RLC phosphorylation level after each ramp velocity. ^*^Significance between all RLC phosphorylation levels at a specific velocity, where *P < *0.05. ^#^Significant difference compared to corresponding phosphorylation levels at 1 FL s^–1^, where *P < *0.05.

Trabeculae with one of three different RLC phosphorylation levels underwent release‐ramp shortening protocols during activation at 20**°**C to a final sarcomere length of 1.94 μm (8% total shortening). Release‐ramp shortening simulates active cross‐bridge cycling at different muscle loads, which would be encountered during systole. At the end of these manoeuvres, force redevelopment occurs in mixed populations of cross‐bridge states, unlike the release protocol, which synchronizes all myosin heads into weakly (or unbound) states. This assay allows us to assess whether changes in *k*_tr_ can be attributed to enhanced thin filament activation by already bound cross‐bridges. The shortening velocities were 1, 0.5 and 0.3 FL s^–1^ (Fig. 5 *A*, *C* and *E*), where 0.3 redevelops force from the highest muscle load with the most attached cross‐bridges, by proportion. For each RLC phosphorylation level, *F*_iso_ was constant across all ramp velocities (Fig. 6 *C*). This was expected because *F*_iso_ was attained at a SL length of 1.94 μm after each manoeuvre velocity. Trabecular preparations with reduced RLC phosphorylation displayed a reduced *F*_iso_ post release (independently of release velocity) of 19 ± 2 kN m^–2^ (*P < *0.05) versus control of 49 ± 4 kN m^–2^ and phosphorylated of 55 ± 3 kN m^–2^ (Fig. 6 *C*).

Normalizing force development at each RLC phosphorylation level for each ramp velocity illustrates the effect of RLC phosphorylation on the rate of force rise (Fig. 5 *B*, *D* and *F*). Trabeculae with control RLC phosphorylation displayed rate constants for force production of 12 ± 1.2, 11 ± 1.1 and 10 ± 0.5 s^–1^ at velocities of 1, 0.5 and 0.3 FL s^–1^ respectively (Fig. 6 *B* and Table 4). Force rose more quickly for trabeculae with increased RLC phosphorylation compared to control (*P < *0.05) at 21 ± 1.6, 19 ± 1.7 and 18 ± 1.6 s^–1^ for velocities of 1, 0.5 and 0.3 FL s^–1^, respectively. Trabeculae with reduced RLC phosphorylation had slower rates of force production compared to control (*P < *0.05) with rates of 10 ± 3.2, 7.6 ± 0.8 and 6.7 ± 1.1 s^–1^ at velocities of 1, 0.5 and 0.3 FL s^–1^, respectively.

**Table 4:** Parameters from exponential fit of release‐ramp protocol

	Phosphorylated (*n = *5)			Control phosphorylation (*n = *5)			Reduced phosphorylation (*n = *5)		
Release velocity (μm)	0.3	0.5	1	0.3	0.5	1	0.3	0.5	1
*F*_iso_	55 ± 3†	54 ± 2†	57 ± 4†	49 ± 4	47 ± 3	50 ± 4	19 ± 2	18 ± 2	17 ± 2
*k*_tr_	18 ± 1.6†	19 ± 1.7†	21 ± 1.6†	10 ± 0.5	11 ± 1.1	12 ± 1.2	6.7 ± 1.1	7.6 ± 0.8	10 ± 3.2

^†^Denotes a significant effect of RLC phosphorylation within a SL length (*P < *0.05).

Clearly, *k*_tr_ is altered by RLC phosphorylation level change at saturating [Ca^2+^] (Fig. 6 *A* and Table 4). The effect is seen for force redevelopment after ramp shortening at all ramp velocities, although the effect of ramp velocity itself is small and does not reach statistical significance. This may infer that additional thin filament activation by cross‐bridge binding is either a small or insignificant effector of *k*_tr_ in this assay, and that changes in *k*_tr_ are associated with mechanisms involving thick filament regulation and not thin filament regulation under these experimental conditions.

## Discussion

RLC phosphorylation is known to be important in many elements of cardiac physiology and disease, including in cardiac developmental organization (Nishio *et al*. 1997; Okamoto *et al*. 2006), in regulation of ventricular torsion (Davis *et al*. 2001; Hidalgo *et al*. 2006), and in acquired (Aoki *et al*. 2000; Gu *et al*. 2010) and inherited diseases (Sanbe *et al*. 1999; Ding *et al*. 2010; Sheikh *et al*. 2012; Warren *et al*. 2012). Of note, the atria and ventricles have separate isoforms of the RLC, and different mammals have different RLC sequences with different numbers of phosphorylatable residues. Accordingly, care needs to be taken when comparing results from different species (Morano, 1992).

We have previously demonstrated the ability of RLC phosphorylation to influence myocardial force production, power and shortening under‐load (Toepfer *et al*. 2013). Previous studies have probed the effect of RLC phosphorylation on *k*_tr_, but have not assessed physiological sarcomere lengths, and have shown conflicting results (Olsson *et al*. 2004; Colson *et al*. 2010). In the present study, we add clarity by showing that, at maximal activation, RLC phosphorylation and, to a lesser extent, sarcomere length alter *k*_tr_. The observed changes are consistent with the Frank–Starling law of the heart and may indicate a new mechanism that contributes to this phenomenon (discussed below). Importantly, our measurements at saturating calcium allow us to consider processes that are independent of the Ca^2+^‐dependent contributions to the Frank–Starling law, and also show that RLC phosphorylation and the thick filament contribution both play a role in the Frank–Starling law.

### The effect of sarcomere length on the rate of force production

Our measurements of *k*_tr_ in control phosphorylation are in the range observed in previous studies at maximal activation between ∼4.5 and 14 s^−1^ with variance as a result of changes in temperature and muscle type (Wolff *et al*. 1995; Fitzsimons *et al*. 2001; Regnier *et al*. 2004). Additionally, *k*_tr_ at control phosphorylation levels (10–11 s^−1^) is close to the ATPase rate in trabeculae from rats under identical experimental conditions (∼8 s^−1^) (Mansfield *et al*. 2012).

Increasing sarcomere length from ∼1.85 to 1.94 μm accelerated *k*_tr_ at each RLC phosphorylation level. The acceleration of *k*_tr_ with stretch, independently of [Ca^2+^], is observed in intact myocardium (Rhodes *et al*. 2015) and is a calcium independent manifestation of length‐dependent activation (de Tombe *et al*. 2010). This is in agreement with the length–tension relationship of trabeculae from rat because the sarcomere lengths used in the present study are in the ascending limb of the length–tension curve (Kentish *et al*. 1986). Some previous studies have shown that *k*_tr_ decreased between SLs of 2.0 and 2.35 μm (Korte & McDonald, 2007; Patel *et al*. 2012) or had no statistical effect (Adhikari *et al*. 2004; Hanft & McDonald, 2010), as well as between 2.0 and 2.2 μm (Milani‐Nejad *et al*. 2013). The differences between these findings and those reported in the present study could conceivably be a result of the SLs assessed or the technique used to assess *k*_tr_ because the aforementioned studies used restretch protocols. The common interpretation of the Frank–Starling law is that, with higher pre‐loads, the myocardium develops a greater systolic force as a result of calcium‐sensitive length‐dependent activation, as well as changes in myofilament overlap (Katz, 2002; de Tombe *et al*. 2010). In accordance with Frank–Starling, our data suggest that the force of contraction increases with pre‐load (an increased stretch of myocardium as a result of increased chamber volume is accompanied by increases in SL). Additionally, our data show that the rate at which force is produced is also affected, although this phenomenon only appears to be evident in situations where RLC phosphorylation is raised or lowered from control levels (Table 3). This phenomenon is beneficial to the ejection fraction because the systolic duration would not need to be prolonged to fully accommodate extra expulsion: *k*_tr_ accelerates to compensate for increased ejection volume. Because this effect is observed at saturating [Ca^2+^], it is independent of calcium‐sensitive activation and the thin filament regulatory complex. Thus, the measured changes in *k*_tr_ are explained by direct interactions between myosin and actin that are independent of thin filament activation and may be a consequence of the increase in *f*, which will alter the distribution of actomyosin states towards strongly‐bound, force‐generating states.

### The effect of RLC phosphorylation on the rate of force production

Our results show that RLC phosphorylation level alters *k*_tr_ over a range of SLs (1.85–1.94 μm) during maximal calcium activation, in contrast to the findings of another study at a higher SL (∼2.3 μm), which observed no change to *k*_tr_ at this higher SL (Olsson *et al*. 2004). The difference in experimental result is probably a result of the aforementioned differences in SL, possibly because *k*_tr_ is influenced by inter filament spacing, which is altered by the length of the muscle (Wang & Fuchs, 1995). Our measurements at the shorter, physiological sarcomere lengths are probably more meaningful during systole (Sonnenblick *et al*. 1967), although saturating [Ca^2+^] and the less than physiological temperature may also influence the ability to extrapolate our findings to the situation in the living animal.

Our measurements are made in saturating [Ca^2+^]; therefore, RLC phosphorylation must be altering cross‐bridge behaviour independently of the thin filament regulatory complex. Consequently, RLC phosphorylation modulates the Frank–Starling law of the heart (although not on a beat to beat basis) (Stracher, 1969). We propose that RLC phosphorylation level is another mechanism by which the myocardium modulates its ability to redevelop force in response to increased ventricular pre‐load.

Interestingly, under chronic raised pre‐load, RLC phosphorylation is raised as a compensatory mechanism to greater ventricular volume (Hidalgo *et al*. 2006), providing further evidence that RLC phosphorylation is an important modulator of systolic ejection in the intact organ. Additionally, experimental models with reduced RLC phosphorylation display increased systolic duration and slowed cross‐bridge kinetics, in accordance with our findings (Abraham *et al*. 2009; Scruggs *et al*. 2009). The use of constitutive RLC pseudophosphorylation to correct RLC phosphorylation reduction has been shown to alleviate this phenomenon (Yuan *et al*. 2015). By contrast, hyperphosphorylation of RLC increases contractility and contractile efficiency independently of any disease phenotype (Huang *et al*. 2008). Together, these findings are complementary to our own. Dephosphorylation of RLC slows *k*_tr,_ which prolongs systole: pre‐load ejection takes longer because systolic contractile kinetics are slowed. Similarly a raised *k*_tr_ as a result of RLC phosphorylation explains increased ejection characteristics as cross‐bridge recruitment is accelerated by RLC phosphorylation.

### Modelling of cross‐bridge attachment (*f*) and detachment (g)

We note that the rate of force redevelopment, *k*_tr_, was well described by fitting a single exponential to the force signal (Brenner, 1986; Metzger *et al*. 1989; de Tombe & Stienen, 2007). This justifies the application of a simple two‐state model to delve into the attachment and detachment kinetics giving rise to the measurable *k*_tr_. In the two‐state model of the cross‐bridge cycle, *k*_tr_ is represented by the sum of the two rate constants, *f* and *g* (Huxley, 1957). Although the two‐state model is a simplistic approximation to cross‐bridge behaviour (Huxley & Simmons, 1971; Podolsky *et al*. 1976), it is a useful tool for exploring the effect of RLC phosphorylation. Modelling of control tissue resulted in values of *f* and *g* that are comparable to previous studies reported in the literature (Brenner, 1988; Prakash *et al*. 1999; Prakash *et al*. 2000). The model provides values for *f*, *g*, DR and *F*_XB_, which describe our experimental findings (Table 3).

The modelling suggests that RLC phosphorylation accelerates *f*, probably as result of a closer myosin head proximity to actin (Levine *et al*. 1996; Colson *et al*. 2010). The model also calculates a modest acceleration of *g* with increasing RLC phosphorylation. Changes to *g* manifest as changes in the ratio between ATPase and isometric force (Huxley, 1957). Indeed, it was shown that myosin light chain kinase treatment increased myosin ATPase and did not alter isometric force, in keeping with this modelling outcome (Muthu *et al*. 2012). At higher RLC phosphorylation, *g* is accelerated and the peak isometric force was stable. The modelling outcome is supported experimentally as the acceleration of *f* must be greater than the acceleration of *g*_,_ otherwise isometric force would fall as phosphorylation increases (Regnier *et al*. 2000). When comparing trabeculae with reduced and increased phosphorylation, the rate of *f* is accelerated ∼20 fold at each SL, whereas *g* is only accelerated ∼1.6 fold. A recent study suggests that RLC phosphorylation slows cross‐bridge detachment (*g*), which does not fit the modelling of our experimental data and contradicts the existing literature (Pulcastro *et al*. 2016). The discrepancy may result from the different experimental and modelling approaches for the study of cross‐bridge kinetics. The lack of experimental records in this publication, as well as the apparent very low isometric force, makes it difficult to explore the source of the discrepancy. Perhaps the phosphorylation state of other sarcomeric proteins is also different from that achieved in the present study (Pulcastro *et al*. 2016).

In our experiments, reduced RLC phosphorylation levels are accompanied by reductions in *F*_iso_ and *k*_tr_. The relative reduction of *F*_iso_ in the trabeculae with reduced RLC phosphorylation compared to control is greater (∼75%) than the reduction in *k*_tr_ (∼30%). This may be accounted for by a shift towards the super relaxed state of the myosin heads (Hooijman *et al*. 2011), reducing *N*. It is probable that either one (or both) of *f* and the myosin ATPase rate must be altered by reduced RLC phosphorylation to account for this reduction of force. Our modelling estimates a marked ∼5‐fold reduction in *f* in the reduced phosphorylation cohort, which, again, is probably explained by myosin proximity to actin (Colson *et al*. 2010).

Our findings cannot rule out the possibility of additional thin filament co‐operativity induced by myosin binding, even in the presence of saturating calcium (Regnier *et al*. 2004). Similarly, acceleration of force redevelopment may well be achieved by other mechanisms that affect *f*, such as the unitary force of each cross‐bridge and the neck region stiffness, or indeed a change in the size of the working stroke under load, which cannot be accounted for by a two‐state model (Nyitrai & Geeves, 2004; Greenberg *et al*. 2009; Karabina *et al*. 2015). Our modelling suggests that *F*_XB_ is not altered by RLC phosphorylation. However, this could be an unavoidable artefact of necessary assumptions in our modelling; namely, *N* remains constant at each RLC phosphorylation level, which we cannot directly address in the present study. However, in skeletal muscle, it was found that *F*_XB_ and *N* were not altered by phosphorylation, which may also translate to cardiac muscle (Sweeney & Stull, 1990). Our calculated value of *F*_XB_ is comparable to that calculated previously in other studies: 1.25–7 pN with an average of ∼3 pN between these studies, which is in good agreement with our approximation from modelling (Ishijima *et al*. 1996; Takagi *et al*. 2006) (Ishijima *et al*. 1996; Nag *et al*. 2015).

A striking finding of our modelling is that SL affected *k*_tr_, *f* and *g*; additionally, stretch influenced the transition from weakly to strongly bound cross‐bridges more than the transition from strongly to weakly bound. This asymmetry suggests a role for lattice spacing effects. Bringing myosin heads closer to actin accelerates *f* at the same time as having a smaller effect on *g*.

The findings of the model reported in the present study are compatible with our previous findings using force velocity and power velocity relations to assess the effect of RLC phosphorylation on cardiac contractile output (Toepfer *et al*. 2013). The finding that RLC phosphorylation accelerates the transition from weakly to strongly bound cross‐bridges is compatible with the observed increase in force and power produced during shortening, as well as with the increase in maximal unloaded shortening velocity seen with enriched RLC phosphorylation (Toepfer *et al*. 2013).

In the context of twitch contractions at lower physiological calcium activations in the myocardium, an increasing RLC phosphorylation may increase the calcium sensitivity of contraction and create greater thin filament activation at lower calcium levels. This would be mediated by co‐operative activation of the thin filament created by RLC phosphorylation, allowing for the faster attachment of cross‐bridges. Additional studies should aim to investigate this effect in submaximal calcium concentrations with this experimental system.

## Conclusions

Under conditions where myosin heads are largely synchronized (post rapid release protocol) or unsynchronized (post release ramp protocol), RLC phosphorylation alters *k*_tr_. SL alters *k*_tr_ on a beat‐to‐beat basis because it is affected by ventricular pre‐load causing myocardial stretching. RLC phosphorylation adds another level of regulation on *k*_tr_, which is observed under chronic conditions of pre‐load, which probably comprises a more long‐term mechanism for fine‐tuning myocardial contractility. In models of disease that are caused by reductions in RLC phosphorylation, the phenotype of ineffectual muscle contraction is brought about by reduced cross‐bridge cycling rates and a reduction in actively cycling cross‐bridges (*N*). Our results complement these findings, highlighting RLC phosphorylation as being a thick filament level regulator of the Frank–Starling law of the heart, which is independent of the thin filament regulatory complex. We suggest that RLC phosphorylation modulates the Frank–Starling law of the heart in both health and disease. It does so by adapting myocardial contractile kinetics, allowing for the timely systolic expulsion of ventricular pre‐load.

## Additional information

### Competing interests

The authors declare that they have no competing interests.

### Author contributions

All data were collected at Imperial College London in the laboratory of Professor Michael A Ferenczi. CNT, TGW and MAF conceived and designed the experiments. CNT and MAF collected and analysed data. CNT, TGW and MAF interpretated data and produced the manuscript.

### Funding

The present study was supported by Wellcome Trust Grant 091460/Z/10/Z.
